# Researcher awareness and submission practices to ethics committees in Saudi Arabia: a cross-sectional study

**DOI:** 10.1186/s12910-026-01382-x

**Published:** 2026-01-17

**Authors:** Roaa S. Bogdadi, Dr. Nahid A. Qushmaq, Eng. Rahaf Al Hasheem, Dr. Marivel M. De Guzman, Wijdan A. Baeshen, Sara M. Aljeaid

**Affiliations:** 1 King Abdullah Medical Complex in Jeddah, Jeddah, Saudi Arabia; 2 At KeyLife Electronics & At Pioneers Academy Jordan-Amman, Jeddah, Jordan; 3 Research Assistant Company, Riyadh, Saudi Arabia; 4 Research Department, Ministry of Health Branch in Jeddah, Jeddah, Saudi Arabia

**Keywords:** Research ethics, Institutional review board, Awareness, Submission practices, Saudi Arabia

## Abstract

Institutional Review Boards (IRBs) and Research Ethics Committees (RECs) are central to safeguarding ethical obligations in research, yet awareness and submission practices among researchers in Saudi Arabia remain inconsistent. This study explored researchers’ awareness, submission methodologies, perceived barriers, and possible improvements to the IRB process. A national electronic cross-sectional survey was distributed to 915 researchers affiliated with academic and research institutions across Saudi Arabia, yielding 870 complete and valid responses (response rate: 95.1%). The questionnaire covered demographics, awareness of ethical principles, submission practices, barriers, and recommendations. Data were examined using descriptive statistics and chi-square tests, with Cramer’s V used to indicate effect sizes. Results showed that prior submission experience was significantly associated with higher awareness of research ethics (87.3% vs. 49.4%, χ²(2) = 149.40, *p* < 0.001, Cramer’s V = 0.414). Perceived institutional support was associated with greater ease of submission (χ²(16) = 195.75, *p* < 0.001, Cramer’s V = 0.237), while formal ethics training was associated with higher confidence in applying ethical protocols (78.2% vs. 48.5%, χ²(1) = 80.32, *p* < 0.001, Cramer’s V = 0.304). Frequently reported barriers included unclear procedures, bureaucratic delays, and limited supervisory guidance, while qualitative analysis highlighted recurring concerns regarding procedural complexity. Despite a well-established regulatory framework, practical gaps in awareness and submission practices persist. Participants recommended structured ethics training, simplified digital submission platforms, and enhanced supervisory engagement to strengthen compliance and reinforce research integrity.

**Supplementary Information:**

The online version contains supplementary material available at 10.1186/s12910-026-01382-x.

## Introduction

The processes of ethical approval are fundamental in safeguarding human participants and securing the reputation of science. The main mechanism for enforcing ethical standards in the research context is the Institutional Review Boards (IRBs) or Research Ethics Committees (RECs). These mechanisms safeguard the conduct of research in an ethical manner, demonstrating integrity, respect, and accountability. Nonetheless, despite the importance of these processes, issues with awareness, compliance, and procedures persist across varying contexts of research. Lack of understanding about ethical processes has been shown to delay obtaining project approval, impair protections for human participants, and jeopardize the reputation of the institution [1]. Recent studies demonstrate that even in well-established research systems, investigators experience annoyance with bureaucratic delays, lack of clarity in submission processes, and inconsistent reviews [2]. These challenges are increasingly linked to limitations in digital ethics infrastructures, such as poorly integrated electronic IRB (e-IRB) platforms, fragmented informatics workflows, and limited user-centered system design, which can further complicate the ethics approval process. These continuing challenges highlight that ethical oversight is not only a matter of policy but also of practice and researcher preparedness, making it a global concern.

Evidence from across the globe demonstrates the universality of these concerns. In the example of South Africa, the new ethics review process revealed ongoing issues with student submissions reflecting lack of preparedness and that subsequently resulted in multiple submissions [3]. In the United Kingdom, researchers have frequently indicated that they viewed IRBs as obstructive rather than facilitating as a result of enforced driving rules and long review times [4, 5]. In addition, several studies have reported that the limited functionality of electronic ethics management systems contributes to inefficiencies, lack of transparency, and user dissatisfaction. Research in China shows sustained gaps in understanding ethics, especially concerning informed consent and data privacy, and early-career researchers reported limited training in official protocols [6]. Researchers in the United States similarly expressed concerns about inefficiencies in multicenter reviews and a lack of harmonization between committees [7]. Likewise, research emerging from the Middle East regarding medical trainees and postgraduate students lessened awareness of fundamental principles of research ethics was associated with inconsistency towards compliance [8]. Together, these studies illustrate that challenges with ethics review are not found in one region, but range across multiple countries and regions into high- and middle-income settings.

With regard to research ethics review, there have been marked improvements in Saudi Arabia in recent years. The National Committee of Bioethics (NCBE) has developed extensive regulations to provide standards for ethical review across institutions, and the majority of universities have their own internal review boards (IRBs). Several institutions have begun implementing electronic IRB submission systems; however, these platforms vary widely in usability, integration, and operational efficiency. Nevertheless, practical challenges remain. Research indicates that even when regulations exist, many researchers, especially students and junior researchers, perceive challenges with respect to ethical duties in their research [9]. Supervisors have different abilities to assist students with developing their proposals, with some students not being able to prepare a proposal without significant help. Coordinators/administrators, who assist students with this work, often have a large bureaucratic burden to do their work, which slows down the process and may decrease the researchers’ motivation. These observations reflect a persistent disconnect between formal regulatory frameworks and the practical realities of navigating ethics review systems, particularly within digitally mediated submission environments.

Although there is international and regional research indicating a lack of awareness and practices related to research ethics, there is little evidence of Saudi researchers’ experiences and their understanding of ethical protocols, submission practices, and barriers. Additionally, only a small number of studies offer perspectives about researchers along career trajectories and across disciplines, leaving to question how the experiences of students and faculty researchers, and research staff may differ. Accordingly, this study aims to systematically investigate researchers’ awareness, submission practices, perceived barriers, and informatics-related challenges within IRB systems across Saudi institutions, and to propose evidence-based recommendations for improving ethics education and electronic submission processes.

## Main objectives

To assess researchers’ awareness, attitudes, and submission practices regarding ethical protocols and IRB requirements in Saudi Arabia.

### Specific objectives

- Assess researchers’ level of awareness of ethical protocols and submission requirements.
- Examine submission practices to ethics committees across different disciplines and career stages.
- Identify the barriers researchers encounter when engaging with ethics committee, including challenges related to digital IRB platforms and informatics workflows.
- Provide recommendations for improving ethics training and submission practices.

## Significance of the study

This study is important as it helps bridge the gap between policy and practice. The work produced from this study will help universities and research organizations strengthen training, streamline submission, and enhance ethical compliance. As a research organization, this is particularly important from a research coordinator’s perspective. Even though students have been formally assigned to a supervisor, inevitably they are often still not ready to write proposals independently, which signifies weak areas of training and mentorship for students. Some researchers also approach ethics compliance as a transactional requirement rather than a scientific responsibility, a perception that undermines the foundational purpose of ethical review. By emphasizing ethics as a core scientific value supported by effective digital infrastructures and researcher-centered IRB systems, this study provides actionable evidence to promote a sustainable culture of integrity, respect, and accountability in Saudi research environments.

## Literature review

The ethical approval process is vital in protecting human participants and ensuring the advancement of trustworthy scientific research. IRBs and RECs are the main strategies by which research will honor ethical guidelines. Despite a global emphasis on ethics, awareness of and practices related to IRBs are inconsistent across settings, resulting in delays, non-compliance and in some cases breaches of ethics. Recent literature increasingly links these challenges to limitations in digital ethics infrastructures, including poorly designed electronic IRB (e-IRB) platforms, fragmented informatics workflows, and lack of system interoperability. In the literature, several repetitive challenges are clearly pertinent to this study, falling into four key areas: (1) difficulties with proposal writing, (2) inadequacies in supervision and training, (3) coordinator and administrative load within IRB processes, and (4) a contrast between intrinsic worth of the science, and extrinsic worth of the research.

### Students’ struggles with research proposals

Evidence suggests that students encounter a number of difficulties in developing research proposals, especially related to the research problem, the approach methodology, and ethical alignment. For example, case studies from Indonesia and Malaysia identified that undergraduate and graduate students had little to no training in proposal writing, and their proposals altogether missed large sections, writing ideas, and had little to no clear meaning [10]. Very similar conclusions were drawn in South Africa, where institutional reviews of student researchers showed students were resubmitting proposals a number of times, mainly due to errors [3]. In digitally mediated ethics systems, such weaknesses are often amplified, as students struggle to navigate complex e-IRB interfaces without adequate instructional support. These issues indicate that students have little to no experience with research ethics during their training programs and are dependent on supervisors to help navigate understanding.

### Weakness in supervision and training

Effective supervision is necessary for assuring ethical and high-quality research. However, shortcomings in supervisory practices are prevalent. It reported that many supervisors do not receive purposeful training in mentoring trainees on ethical or scholarly projects to adapt to funding agency regulations [11]. A pilot study of supervisor training demonstrated that if supervisors were not trained systematically, they could unintentionally transmit poor practice that results in insufficiency to comply with ethical standards [12]. Notably, studies in higher education noted that gaps in supervisor training are influential in inconsistent quality of student proposals, and in breaching a mutual agreement to be ethical participants [5]. These supervisory deficits are particularly problematic in institutions using electronic submission systems, where supervisors are expected to guide students through complex digital workflows without standardized training. Findings also correspond to the barriers in the present study where postgraduate students frequently reported not having enough support from their supervisors was a primary barrier in figuring out the submission process to the funding agency [11, 12].

### Coordinator and administrator burdens in IRB systems

Institutional Review Boards (IRBs) are an important component of maintaining ethical practices, while the coordinators and administrators engaged with these processes are often expected to work out convoluted, often bureaucratic, processes. For example, in South Africa, research has reported that ethics committees were routinely criticized for slow timelines for review, vague or ambiguous requirements, and burdensome administrative requirements [3]. Similarly in the U.K., educational researchers noted that ethics committees were sometimes viewed as “foes” rather than allies due to overly constituted instances of guidance and variability in the guidance [4, 5]. The realities associated with multi-institutional studies are even more complex. Klitzman described the interpretation of local knowledge as an amalgam to the inconsistencies in interpretation that delineated a structural concept for review [13], while Stommel and Rijk provided of an example of online researchers not only having to address continuing changes but also having to reconcile changes with currently approved forms [7]. From an informatics perspective, these issues are exacerbated by poorly integrated e-IRB systems that lack automation, real-time feedback, and interoperability across institutions, thereby increasing administrative workload and approval delays.

### Incentives versus intrinsic scientific valueInce

Another recurrent point in the literature is the influence of incentives on the behavior of researchers. Studies suggest that institutional pressure, such as promotion, funding, and recognition, underpin compliance with research ethics, as opposed to internal values about being a “good scientist” [14, 15]. Cheung et al. further remarked that supervisors might encourage productivity instead of integrity, which only serves to reinforce extrinsic motivations [11]. However, other scholars suggested that quality research occurs when ethics is positioned as part of scientific integrity—an accomplishment within itself rather than a transactional requirement [1]. This tension is particularly evident in digital IRB environments, where ethics compliance is often reduced to a checklist-driven task rather than a reflective scientific process. From a research coordinator’s perspective, there is a need to re-frame ethics as part of scientific integrity in which compliance is pursued, not for external reward, but to ensure protection of participants and means to promote knowledge.

### Research gap

Although studies internationally from Jordan, China, South Africa, the UK, and the US have examined lack of researcher awareness, supervision, and institutional systems, few studies have investigated this in the Saudi context. Furthermore, no study has examined the differences between students, faculty, and coordinators in a systematic way to examine awareness of research ethics, submission practices, barriers, and recommendations related to the ethics of research. This study addresses this gap by integrating quantitative and qualitative evidence from Saudi institutions, with particular emphasis on experiences within electronic IRB workflows and digitally mediated ethics review systems.

## Methods

### Research design

This study employed a cross-sectional descriptive survey design to assess researchers’ awareness, attitudes, and submission practices regarding Institutional Review Board (IRB) requirements and research ethics. The study was conducted in Saudi Arabia, and data collection took place between 1 June to 30 August 2025.

### Participants

A total of 915 researchers representing Saudi academic and research institutions received invitations to the participation, using a national institutional-based sampling approach that targeted universities, medical colleges, and research centers across multiple regions of Saudi Arabia. Of those, 870 researchers responded yielding a high response rate of 95.1%. The targeted to study included faculty members, post-graduate students, and research staff working with human subjects. Inclusion criteria specified that participant be currently engaged in conducting research during the time of the study and researchers who were not conducting human subject research were excluded.

### Instrument

Data were collected through a structured self-administered questionnaire which was developed by the research team in accordance with institutional policy and existing literature [3, 9]. The questionnaire consisted of three sections; (1) demographic and academic characteristics, (2) awareness of ethical principles and submission requirements, and (3) attitudes and practices related to IRBs/RECs. Content validity was established through expert review by three specialists in research ethics and IRB governance. Minor revisions were implemented following their recommendations. The questionnaire was pilot-tested among 25 researchers to ensure clarity, relevance, and usability, and internal consistency was assessed using Cronbach’s alpha (α = 0.82), indicating acceptable reliability. A total of 870 valid responses were used in the analysis, and all tables and figures use this number as the denominator. The final English version of the questionnaire is provided in Supplementary File 1.

### Data collection procedure

The survey was distributed electronically through institutional mailing lists and professional research groups, and responses were collected over a three-month period. Participation was voluntary, and informed consent was obtained prior to beginning the survey. No personal identifiers were collected to ensure participant anonymity.

### Data analysis

The analysis of data was carried out using Python (version 3.11) and the pertinent statistical libraries (e.g., pandas, scipy and statsmodels). Descriptive statistics were created to summarize demographics queries and outcome measures. Chi-square tests were employed to test associations between categorical variables and significance was set at *p* < 0.05. Effect sizes were interpreted using Cramer’s V, classified as small (0.10–0.29), moderate (0.30–0.49), and large (≥ 0.50). Missing data were minimal (< 2%) and handled using complete-case analysis. Assumptions for chi-square testing, including minimum expected cell counts, were verified prior to analysis.

### Qualitative analysis

Open-ended responses were analyzed using an inductive content analysis approach. Two independent researchers initially reviewed the responses and developed a preliminary coding framework based on recurring concepts. The coders independently applied the coding scheme to all responses, after which discrepancies were discussed and resolved through consensus. Inter-rater reliability was assessed using Cohen’s kappa, yielding a value of 0.86, indicating high agreement. Final categories were used to generate word-frequency outputs and the word cloud visualization.

### Limitation

Although the questionnaire demonstrated acceptable content validity and internal consistency, construct validity testing and factor analysis were not conducted, representing a limitation of the study.

### Ethics approval

This study was reviewed and approved by the MOH Research Ethics Committee, Jeddah, Saudi Arabia (Approval No. A02221; Approval Date: 29 May 2025). The study adhered to the Declaration of Helsinki. Participation was voluntary, electronic informed consent was obtained, and responses were anonymized.

## Results

A total of 870 valid responses were analyzed. The results below present the key associations among institutional support and perceived submission ease, submission experience and awareness, and training and confidence (see Table 1). The sample consisted of faculty members, postgraduate students, and research staff from multiple disciplines, providing an overall representation of researchers from academic institutions in Saudi Arabia. Of the 870 participants, 702 (80.7%) reported previous IRB/REC submission experience.

**Table 1:** Cross-tabulation between institutional support for ethics submissions and ease of submission process

Institutional Support	Very Difficult	Difficult	Neutral	Easy	Very Easy	Row Total
Strongly Disagree	35.5%	19.4%	35.5%	6.5%	3.2%	100%
Disagree	10.8%	37.3%	45.8%	6.0%	0.0%	100%
Neutral	5.4%	25.1%	66.2%	3.0%	0.3%	100%
Agree	1.9%	11.8%	62.7%	20.8%	2.8%	100%
Strongly Agree	3.1%	15.6%	50.0%	18.8%	12.5%	100%

The chi-square test revealed a statistically significant association between institutional support for ethics submissions and the perceived ease of the submission process, χ²(16) = 195.75, *p* < 0.001, with a small effect size (Cramer’s V = 0.237). Table 1 presents row percentages and corresponding raw frequencies (N). Row percentages were calculated so that the total across each row equals 100%, allowing comparison of how respondents within each level of institutional support distributed their responses regarding the ease of submission.

For instance, among those who strongly agreed that their institution provides sufficient support, only 3.1% reported that the process was very difficult, 15.6% reported that it was difficult, 50.0% said it was neutral, 18.8% said it was easy, and 12.5% said it was very easy. In contrast, those who strongly disagreed with the statement were over-represented in the “very difficult” category (35.5%).

These findings indicate that perceived institutional support is associated with perceived ease of IRB submission, but do not imply causality. The magnitude of the association was small (Cramer’s V = 0.237), indicating a statistically significant but modest relationship between the variables.

### Awareness of ethical protocols

Overall, researchers showed moderate degrees of awareness of ethical principles and submission requirements (see Table 2). Of the 870 participants, 630 (72.4%) reported being aware, 88 (10.1%) were not aware, and 152 (17.5%) were unsure of their institution’s ethics committee procedures. Many were able to state the purpose of the IRB review process and recognized the need for an individual to provide informed consent prior to involvement in research with human subjects. However, there were significant gaps around data confidentiality, multicenter studies, and particular procedural requirements for submission.

**Table 2:** Association between submission experience and awareness of research ethics

Submission Status	Aware *n* (%)	Not Aware *n* (%)	Not Sure *n* (%)	Row Total
Not Submitted	169 (49.4%)	65 (19.0%)	108 (31.6%)	342
Submitted	461 (87.3%)	23 (4.4%)	44 (8.3%)	528
Total	630 (72.4%)	88 (10.1%)	152 (17.5%)	870

**Fig. 1:**
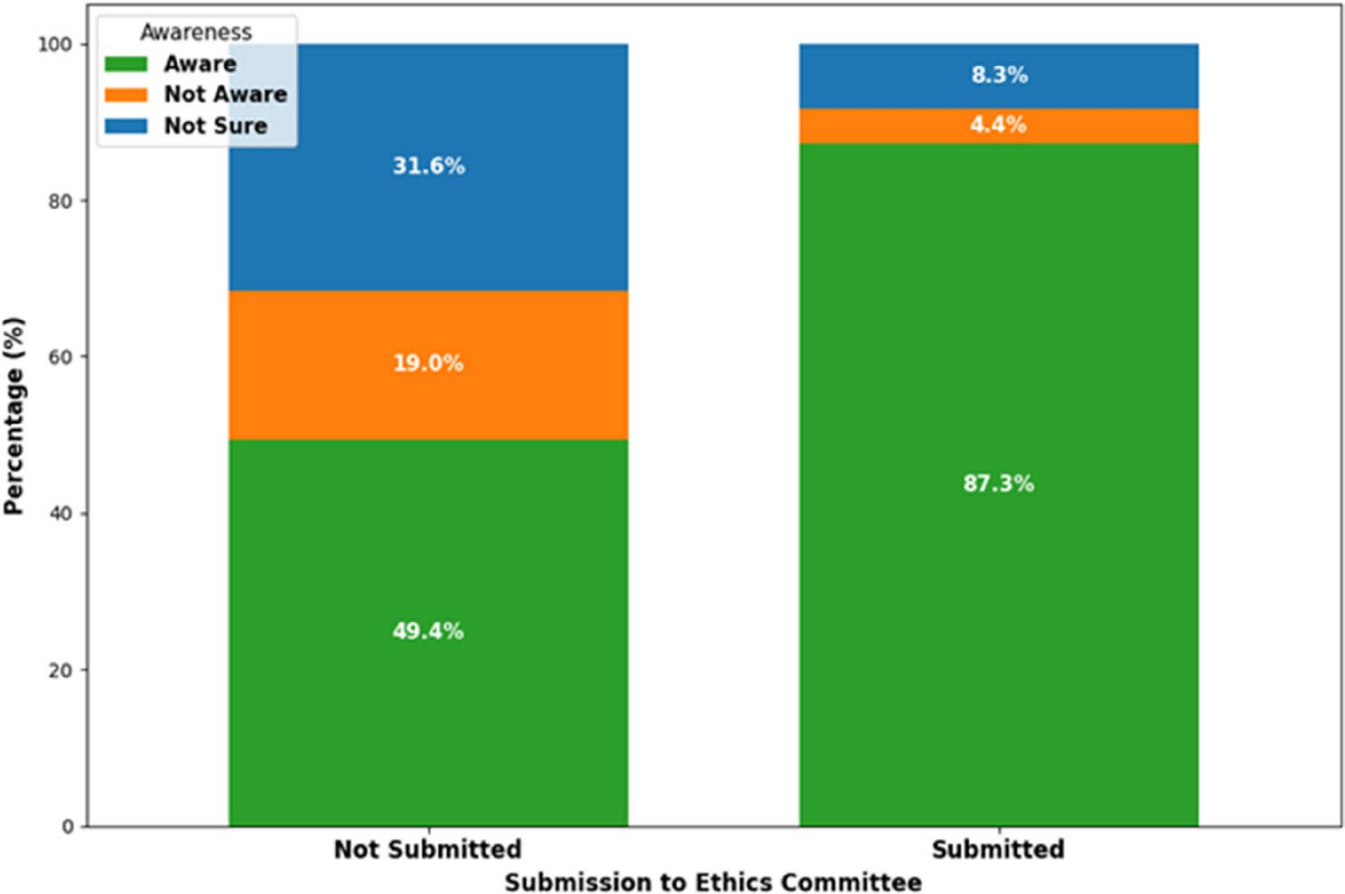
Relationship between submission to an ethics committee and awareness of the committee

A chi-square test of independence identified a statistically significant association between submission experience and awareness of research ethics (χ²(2) = 149.40, *p* < 0.001). Among researchers who had previously submitted protocols (*n* = 528), 461 (87.3%) were aware, 23 (4.4%) were not aware, and 44 (8.3%) were unsure of IRB procedures. In contrast, among those who had not submitted (*n* = 342), only 169 (49.4%) were aware, while 65 (19.0%) were not aware and 108 (31.6%) were unsure (see Fig. 1).

These findings indicate that prior submission experience is associated with higher reported awareness of ethics committee procedures, but do not establish a causal relationship.

Figure 1 visually summarizes the distribution of awareness levels (“Aware,” “Not Aware,” and “Not Sure”) according to submission status. The stacked bar chart demonstrates that researchers who had previously submitted protocols were more frequently categorized as aware of institutional ethics committee procedures, while non-submitters were more likely to report lack of awareness or uncertainty. This figure provides a graphical representation of the association reported in Table 2.

Patterns of submission varied according to academic status and discipline (Table 3). Faculty members were more likely than postgraduate students to have previously submitted research protocols, whereas many postgraduate students reported limited or no submission experience. For example, respondents from biomedical disciplines reported higher rates of prior IRB submission compared with those from social science disciplines. Approximately half of respondents described the submission process as challenging, most commonly citing unclear requests, prolonged review timelines, and difficulties in obtaining advance institutional approvals and informed consent documentation. These findings indicate that submission practices are associated with both career stage and disciplinary context, without implying causal relationships.

**Table 3:** Association between research ethics training and confidence in Understanding ethics protocols

Training Status	High Confidence	Low Confidence	Row Total
Not Trained	162 (48.5%)	172 (51.5%)	334
Trained	419 (78.2%)	117 (21.8%)	536
Total	581	289	870

*Chi-square test*: χ²(1, *N* = 870) = 80.32, *p* < 0.001

*Effect size*: Cramer’s V = 0.304 (moderate effect)

### Submission practices across career stages and disciplines

Patterns of submission varied considerably depending on academic status and discipline (see Table 3). Faculty members were more likely than postgraduate researchers to have submitted research protocols previously, and many postgraduate researchers reported little or no experience submitting protocols to an IRB. For instance, biomedical research reported higher rates of submission than social science research, which highlighted discipline-specific research cultures and ethics oversight. About half of respondents indicated that they found submitting research protocols “challenging” and often named unclear requests, slow review processes, and obtaining approval from advance notice through institutional informed consent obtaining as challenging aspects of the submission process. These findings correspond with our second goal in demonstrating the ways that researcher submission practices differ based on academic status and discipline.

### Association between research ethics training and confidence

**Fig. 2:**
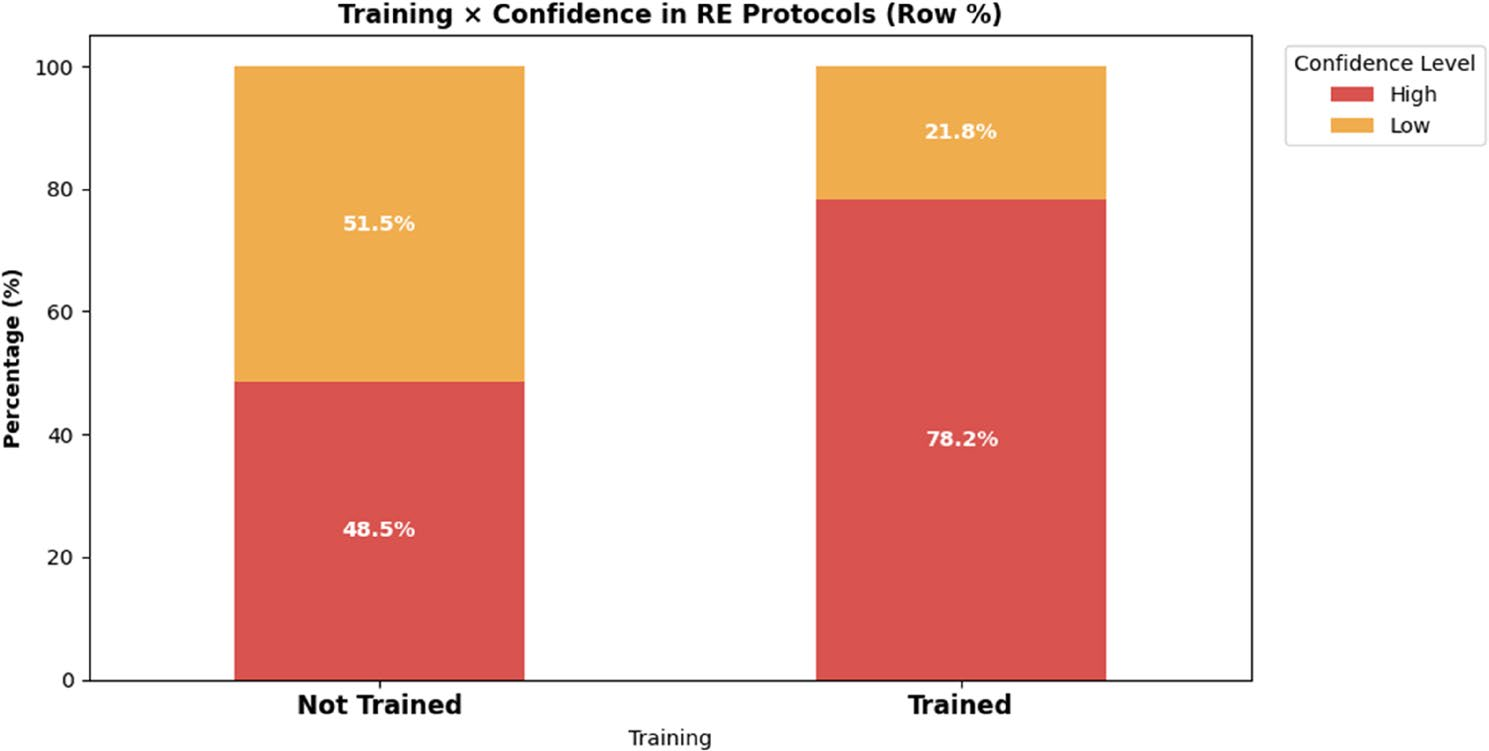
Association between research ethics training and confidence in RE protocols

The analysis indicated a statistically significant association between receiving formal research ethics training and confidence in understanding ethics protocols. Among respondents who reported not receiving training (*n* = 334), 48.5% (*n* = 162) reported high confidence, while 51.5% (*n* = 172) reported low confidence. In contrast, among respondents who reported having received training (*n* = 536), 78.2% (*n* = 419) reported high confidence, whereas 21.8% (*n* = 117) reported low confidence (see Fig. 2).

This association was statistically significant (χ²(1) = 80.32, *p* < 0.001), with a moderate effect size (Cramer’s V = 0.304), indicating that research ethics training is associated with higher self-reported confidence without implying causality.

### Barriers to engaging with ethics committees

A statistically significant association was observed between awareness of the ethics committee and prior submission of research protocols. Among respondents who reported being aware of the committee (*n* = 630), 461 (73.2%) had previously submitted at least one protocol, while 169 (26.8%) had not. In contrast, among those who were not aware of the committee (*n* = 88), only 23 (26.1%) reported having submitted a protocol, whereas 65 (73.9%) had not. Participants who were uncertain about the existence of an ethics committee (*n* = 152) demonstrated similarly low submission rates, with 44 (28.9%) reporting prior submission and 108 (71.1%) reporting none (see Table 4).

**Table 4:** Association between awareness of the ethics committee and submission of research protocols

Awareness of the Ethics Committee	Not Submitted	Submitted	Row Total (%)
Aware (*n* = 630)	169 (26.8%)	461 (73.2%)	100%
Not Aware (*n* = 88)	65 (73.9%)	23 (26.1%)	100%
Not Sure (*n* = 152)	108 (71.1%)	44 (28.9%)	100%
Total (*N* = 870)	342 (39.3%)	528 (60.7%)	100%

*Chi-square test*: χ²(2) = 149.40, *p* < 0.001

*Effect size*: (Cramer’s V) *=* 0.414 (medium-to-strong effect)

The chi-square test indicated that this association was statistically significant (χ²(2) = 149.40, *p* < 0.001), with a moderate-to-strong effect size (Cramer’s V = 0.414). These findings indicate that awareness of the ethics committee is associated with submission behavior, without implying causality.

**Fig. 3:**
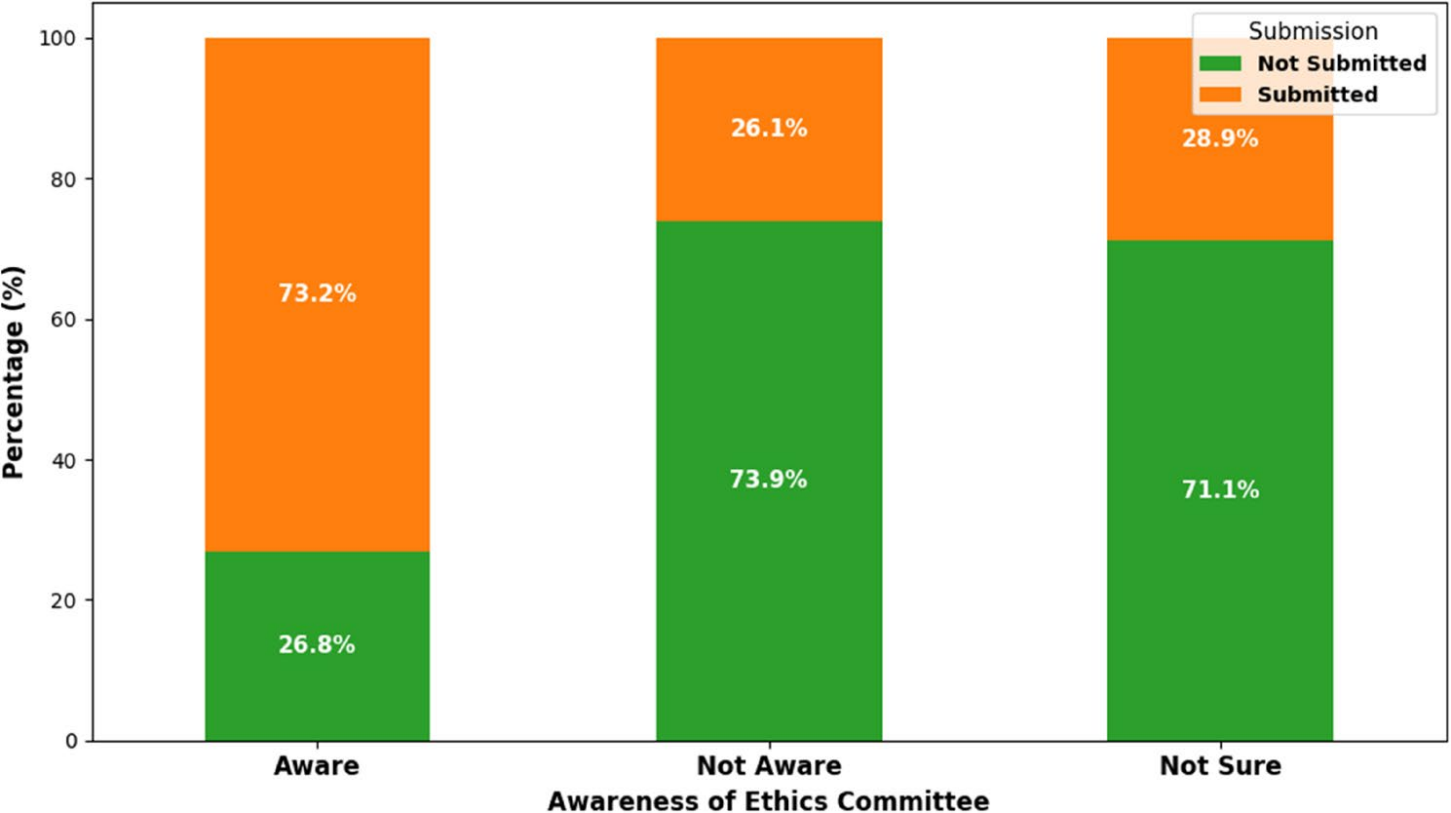
Awareness of the ethics committee and the likelihood of submitting a protocol

Row percentages were calculated such that each awareness group would total 100%, which meant that row percentages reflect the distribution of submission behavior in each group. For example, among those participants who were aware of their institution’s ethics committee, 73.2% had submitted at least one protocol, while for 26.8%, submission did not occur. In the “Not Aware” group, 26.1% had submitted while 73.9% had not, while in the “Not Sure” group, 28.9% had submitted compared with 71.1% who had not (see Fig. 3). This method facilitates comparison across groups without implying that 100% represents the entire sample.

**Fig. 4:**
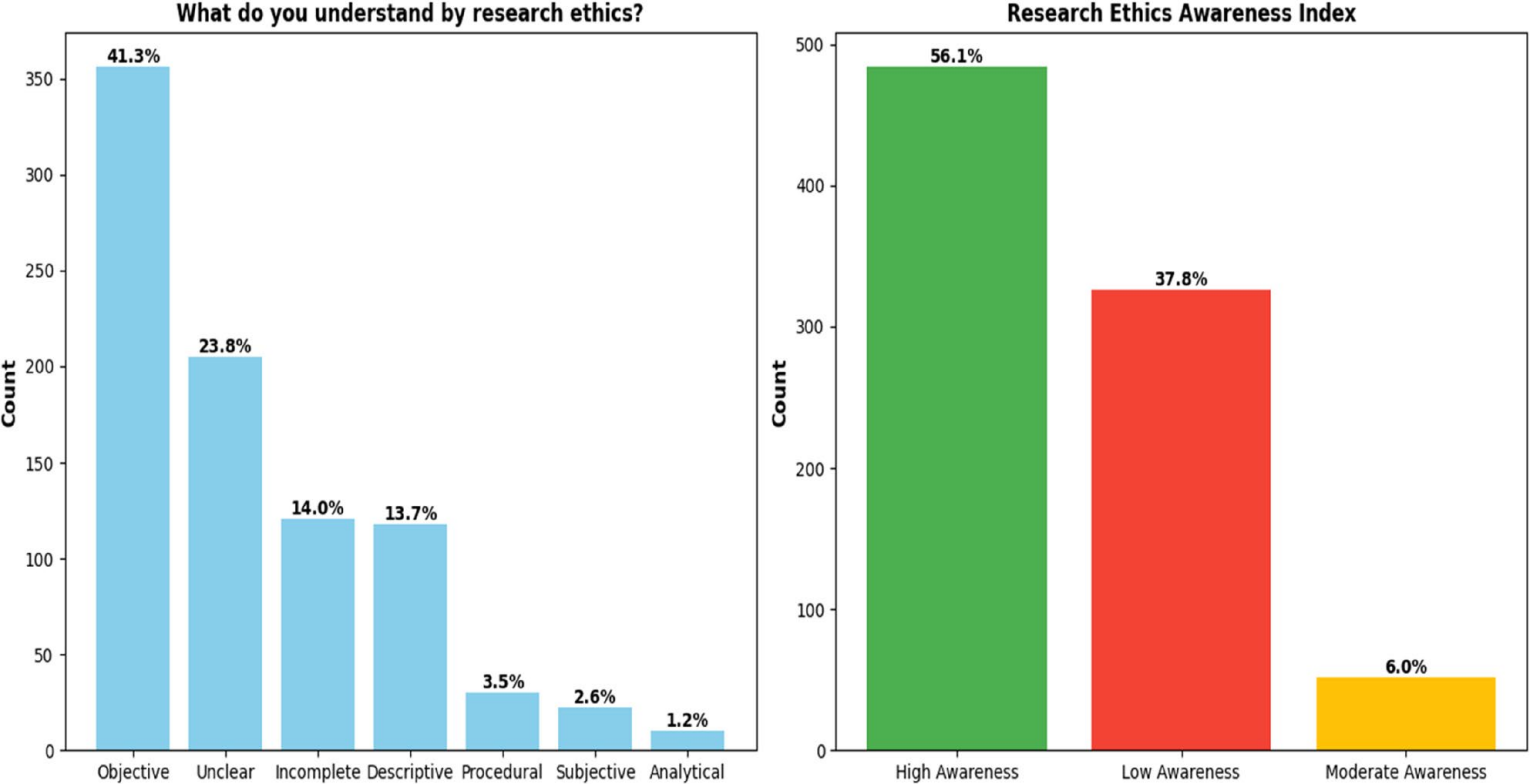
Open-ended definitions of research ethics and overall awareness index among participants

These findings highlight that institutional awareness is a critical facilitator of engagement with IRBs/RECs. Researchers who are unaware—or uncertain—are much less likely to follow submission procedures. This indicates that gaps in communication within an institution function as structural barriers, and add to other challenges such as limited training; bureaucratic delays; and insufficient support from mentors. Addressing these barriers in a comprehensive way is key for enacting ethical compliance at varying levels of academia.

(A) Distribution of responses to the open-ended question “What do you understand by research ethics?” Responses were manually categorized into seven groups. The majority provided objective definitions (41.3%), such as emphasizing the protection of participants or the integrity of procedures. A considerable proportion gave unclear (23.8%) or incomplete responses (14.0%), while descriptive (13.7%), procedural (3.5%), subjective (2.6%), and analytical (1.2%) answers were less common (see Fig. 4). These findings indicate that although many researchers demonstrate a clear understanding of research ethics, a sizeable proportion continue to express vague or partial conceptions, reflecting heterogeneity in ethical awareness across the sample.

(B) Ethics Awareness Index is a combined record of different elements of ethics awareness. More than half of the participants demonstrated high awareness (56.1%), whereas 37.8% were classified as having low awareness, and only 6.0% showed moderate awareness. This suggests a polarization in the sample: most either have a strong understanding of ethics or a limited one, with few in-between.

#### Qualitative insights into research ethics definitions

**Fig. 5:**
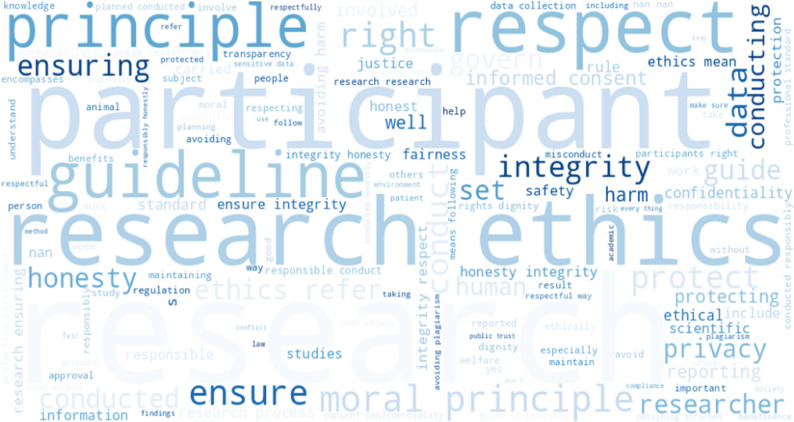
Word cloud of the most frequent terms in participants’ definitions of research ethics

In addition to closed-ended survey items, participants were asked to provide open-ended definitions of research ethics. They analyzed the contents of their responses through a word frequency approach which was then portrayed as a word cloud (see Fig. 5). This qualitative analysis is representative of the key terms focused on by respondents, providing a more nuanced perspective of how researchers conceptualize what research ethics is in a personal sense beyond the established questionnaire items.

The word cloud illustrates the frequency distribution of terms found in respondents’ open-ended definitions of research ethics. The frequently mentioned terms included “research,” “ethics,” “participants,” “principle,” “guideline,” “respect,” and “integrity,” highlighting the themes and topics prioritized by respondents. The size of the word corresponds to its frequency, meaning a larger term is observed more frequently in the dataset. Also, notable terms that emerged included “privacy,” “protection,” “confidentiality,” and “harm,” which shows the respondents are aware of the rights of participants and protection of data. This visualization represents qualitative insight into researchers’ conceptualizations of research ethics, mostly highlighting principles, respect, and protection of participants.

## Discussion

This study investigated researchers’ awareness, submission patterns, barriers, and recommendations regarding ethical protocols in Saudi institutions. The findings indicate variations in awareness, irregular submission patterns across different career stages and disciplines, as well as systemic obstacles to compliance. The findings align with international literature and underscore distinct institutional challenges in Saudi Arabia.

### Awareness of ethical protocols

Researchers with prior submission experience reported substantially higher awareness of ethics committee procedures, while uncertainty was more prevalent among those without submission experience. These results are consistent with findings from Jordan, South Africa, and China, where early-career researchers commonly demonstrate limited understanding of IRB processes and confidentiality requirements [3, 6, 8]. Similar inconsistencies in awareness have also been documented in high-income settings, including the United Kingdom and United States [4].

These patterns suggest that direct exposure to ethics submission workflows is associated with greater awareness; however, reliance on experiential learning alone may disadvantage students and junior researchers who have not yet navigated these systems.

### Submission practices across career stages and disciplines

The practices in submissions exhibited substantial variability between disciplines and career stages. Compared to postgraduate students and social science researchers, faculty and biomedical researchers were far more likely to have submitted a proposal. These differences are somewhat indicative of disciplinary research cultures, notably in the UK and United States where biomedical researchers tend to enforce ethics training more prescriptively than those in social branches [5, 7]. Trained researchers consistently reported greater confidence in understanding ethics protocols compared with those who had not received training. Similar patterns have also been reported in Korea and Europe, where ethics training was associated with higher levels of compliance and lower rejection rates [13, 16].

These findings emphasize the role of institutional training cultures and disciplinary norms in shaping ethics engagement.

### Barriers to engaging with ethics committees

Participants perceived inadequate training, time delays driven by bureaucracy, and lack of oversight and supervision as significant obstacles. Our findings align with previous reports from Africa and the Middle East, indicating that ethics committees encounter challenges related to slow review processes and limited capacity [3, 17]. In multisite studies, response times, variations across sites, and fragmentation of the research ethics boards also present enormous challenges [13].

Such barriers appear to disproportionately affect postgraduate students, who reported lower confidence and greater difficulty navigating submission requirements.

### Qualitative insights into research ethics definitions

The analysis of open-ended responses and the word cloud revealed a recurring theme characterized by terms such as “ethics,” “participants,” “respect,” and “integrity,” alongside references to “privacy,” “protection,” and “confidentiality.” The findings align with research from Canada and the UK, indicating that while researchers articulated their moral values in defining ethics, they lacked clarity on the implementation of ethical practices [5, 17]. Respondents often demonstrated limited procedural clarity regarding implementation of these principles. This gap between conceptual understanding and operational practice underscores the need for clearer guidance within IRB systems.

### Recommendations for improvement

Both the participants consistently suggested the need for formal training in ethics, easier digital platforms, and an increase in engagement from supervisors. These suggestions are consistent with the conclusions from studies conducted in both China and Australia, which found the presence of a digitized submission system resulted in reduced administrative burden and improved transparency and accountability [4, 6]. Supervisor training has similarly been emphasized by researchers in Europe and North America where supervisors, as mentors, are seen as ethical role models for their students [11].

Collectively, these findings support the need for institutional strategies that integrate structured ethics education with effective digital IRB infrastructures to promote consistent, informed engagement with ethics committees.

## Conclusion

This study found that although Saudi institutions have institutional review boards, awareness of and submission practices related to research ethics continue to be inconsistent, especially amongst postgraduate students and early-career researchers. The results indicated that experience with submissions and formal training are associated with higher levels of awareness and confidence while barriers such as insufficient training, bureaucratic delays, and inadequate supervision continue to inhibit compliance. Qualitative responses also indicated that while researchers value principles such as integrity, respect toward participants, and protection of participant safety, many of them are unable to translate their perspective into practices when making submissions.

The recommendations made by participants—structured ethics training, improved digital submission processes, and engagement of supervisors—demonstrated local relevance and international best practices. Addressing these issues may help close the gap between policy and practice in ways that allow ethics review processes to be acknowledged as contributing to ethical practices, rather than bureaucratic obstacles to conducting research.

As a research coordinator, the findings speak to the need for a research culture where the normative ethical practice is upheld as a scientific value, rather than done to garner prestige or as an external motivation while being a relatively rare occurrence. Universities and research institutions should prioritize the implementation of systematic education on research ethics, provide mentorship to students, and promote a transparent submission system to review bodies that facilitates meaningful interaction with the ethical review process. Upholding ethical research practices supports the protection of research participants’ well-being, enhance compliance, and maintain the overall quality and credibility of research.

### Recommendation

This study’s findings lead to several practical recommendations to enhance awareness of ethics and submission to ethics committees in Saudi institutions and other contexts.

1. Institutionalize Research Ethics Training at All Career Stages

Ethics training should be mandatory for all undergraduate, postgraduate, and early-career researchers. Workshops that are scheduled regularly, combined with curriculum elements in ethics, would serve to strengthen awareness of ethical principles, conditions for informed consent, and data protection, to a level where issues related to matters are not left to an individual’s responsibility but systematically built into the development of researchers.

2. Develop Easier Digital Submission Platforms

Timely, transparent online systems may help reduce delays in processing, as well as engage researchers more effectively with ethics committees. As witnessed in studies in China and Australia, ethics management through digitized systems has been associated with improved efficiencies, accountability, and consistency (Zhang, et al., [6]; Brown, [4]).

3. Increase Supervisor Training and Mentorship

Supervisors are an effective means of supporting their students in research proposal preparation and submission of ethical approval. Robust supervisor training programs would be a requirement to ensure that supervisors act as ethical models of the responsible practices of researchers (as recommended in Europe and North America) (Cheung, et al., [11]).

4. Promote a Culture of Ethics as Scientific Integrity, Not Bureaucracy

Ethics should be embraced as a safeguard for participants and a marker of scientific integrity rather than a procedural barrier or a pathway to recognition. Cultivating this mindset requires institutional commitment, leadership support, and the active involvement of research coordinators in bridging policy and practice.

Implementing these measures may support efforts to address the identified gaps, reduce barriers to ethical compliance, and enhance the credibility and integrity of scientific research.

## Supplementary Information

Supplementary Material 1.

## Data Availability

The datasets used in this study is available upon request to the corresponding author. The full English version of the validated questionnaire used in this study is available in Supplementary File 1.
