# Supplementary material for: Researcher awareness and submission practices to ethics committees in Saudi Arabia: a cross-sectional study
Source: BMC Med Ethics. 2026 Jan 17;27:21. doi: 10.1186/s12910-026-01382-x (PMC12895601; doi:10.1186/s12910-026-01382-x)
Supplement: Supplementary file 1 — Supplementary Material 1. [file 12910_2026_1382_MOESM1_ESM.docx]

Understanding Ethical Protocols: A Study on Researcher Awareness and Submission Practices to Ethics Committees

**Consent to Participate in a Research Study**

You’re being asked to take part in a research study that looks at how researchers understand and interact with ethics committees and the processes involved in submitting research for ethical approval.

If you agree, you’ll complete an online survey that takes about [10 M] minutes. The questions focus on your experience with ethical submissions, your understanding of the process, and your opinions.

If you start the survey and change your mind, you can stop at any time. There will be no penalty for not participating or withdrawing.

The survey is anonymous. We won’t ask for your name or any identifying details. All responses will be stored securely and used only for research purposes.

There are no known risks to taking part. While there’s no direct beneﬁt to you, your input will help us understand and possibly improve how ethical practices are handled in research settings.

If you have any questions about the study, feel free to reach out to [**Roaa . S . Bogdade**] at [[**roaathre@gmail.com**](mailto:roaathre@gmail.com)].

* Indicates required question

1. **You voluntarily agree to participate in this study.** *

*Mark only one oval.*


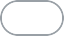
 I consent to participate in this survey. *Skip to question 2*


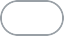
 I am not consent to participate in this survey. *Skip to question 41*

Section 1: Demographics

1. **Gender** *

*Mark only one oval.*


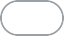
 Male
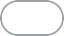
 Female

1. **Nationality** *

*Mark only one oval.*

Saudi
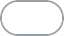
 Non-Saudi


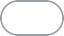


1. **What is your current position?** *

*Mark only one oval.*

Undergraduate Student Graduate Student Research Assistant Lecturer

Professor Doctor Nurse Pharmacist

Medical Assistant Lab Technician Radiologist Physiotherapist Administrator

Other:

1. **What is your ﬁeld of research?** *

*Mark only one oval.*


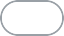
 Natural Sciences


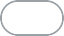
 Engineering & Technology
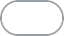
 Medical & Health Sciences
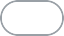
 Social Sciences

Humanities


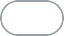


1. **How many years of research experience do you have?** *

*Mark only one oval.*


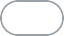
 Less than 1 year
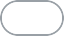
 1_3 years


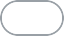
 4_6 years
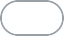
 7_10 years


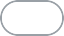
 More than 10 years
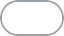
 No experience.

1. **Work Aﬃliation:** *

*Mark only one oval.*

Government Hospital. University.

Private Hospital/Clinic. Research center.

Other:

Section 2: Awareness of Ethical Protocols

1. **Are you aware of your institution's research ethics committee?** *

*Mark only one oval.*


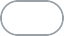
 Yes
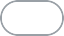
 No


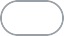
 Not Sure

1. **Have you received any formal training on research ethics?** *

*Mark only one oval.*


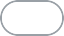
 Yes
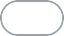
 No

1. **How conﬁdent are you in your understanding of research ethics protocols?** *

*Mark only one oval.*


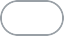
 Not at all conﬁdent.
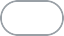
 Slightly conﬁdent.
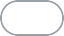
 Moderately conﬁdent.
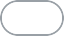
 Very conﬁdent.


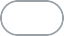
 Extremely conﬁdent.

Section 3: Submission Practices

1. **1.Have you ever submitted a research proposal to an ethics committee?** *

*Mark only one oval.*


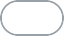
 Yes *Skip to question 12*


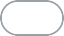
 No *Skip to question 13*

1. **If yes, how many times have you submitted?** *

*Mark only one oval.*


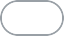
 1 time
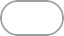
 2_3 times
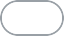
 4_5 times


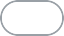
 More than 5 times
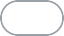
 Not applicable

What type of research required ethics approval?

1. **2.What type of research required ethics approval?** *

*Mark only one oval.*


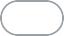
 Human subjects research
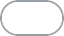
 Animal studies


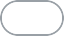
 Secondary data analysis
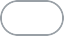
 Not applicable

1. **3.How would you rate the ease of the ethics committee submission process?** *

*Mark only one oval.*


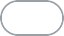
 Very Diﬃcult
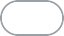
 Diﬃcult
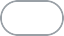
 Neutral
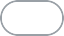
 Easy


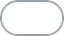
 Very Easy

Section 4: Institutional Support

1. **1.Does your institution provide adequate support for ethics submissions?** *

*Mark only one oval.*


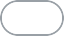
 Strongly Disagree
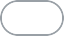
 Disagree


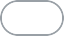
 Neutral
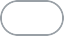
 Agree


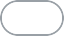
 Strongly Agree

1. **2.Do you feel there is a need for more training or guidance on ethics protocols at** *

your institution?

*Mark only one oval.*


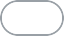
 Strongly Disagree
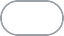
 Disagree


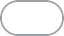
 Neutral
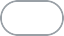
 Agree


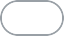
 Strongly Agree

Section 5: Attitudes and Beliefs

1. **1.Ethics review processes are essential to protect research participants.** *

*Mark only one oval.*


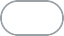
 Strongly Disagree
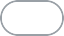
 Disagree


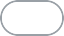
 Neutral
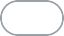
 Agree


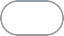
 Strongly Agree

1. **2.Ethics protocols are a barrier to eﬃcient research.** *

*Mark only one oval.*


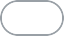
 Strongly Disagree
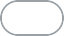
 Disagree


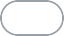
 Neutral
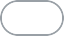
 Agree


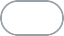
 Strongly Agree

1. **3.I would feel comfortable reaching out to the ethics committee for clariﬁcation.** *

*Mark only one oval.*


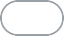
 Strongly Disagree
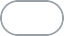
 Disagree


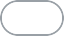
 Neutral
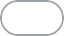
 Agree


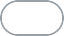
 Strongly Agree

Section 6: Compliance and Monitoring

1. **1.How often do you review ethical guidelines relevant to your ﬁeld?** *

*Mark only one oval.*


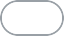
 Never


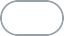
 Once a year
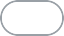
 Twice a year


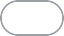
 Every new project


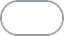
 Continuously / Frequently

1. **2.Have you ever had a project ﬂagged for non-compliance with ethical** *

standards?

*Mark only one oval.*


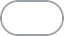
 Yes *Skip to question 22*


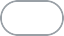
 No *Skip to question 23*


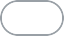
 Not Sure *Skip to question 23*

If yes, what was the main issue? (Only show if previous answer is “Yes”)

1. **If yes, what was the main issue?** *

*Mark only one oval.*


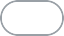
 Incomplete documentation
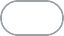
 Participant consent issues


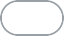
 Privacy/data protection concerns
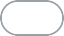
 Unauthorized modiﬁcations


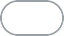
 Other

Does your institution conduct periodic ethics audits or reviews?

1. **3.Does your institution conduct periodic ethics audits or reviews?** *

*Mark only one oval.*


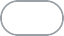
 Yes
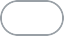
 No


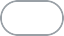
 Not Sure

Section 7: Digital and Data Ethics

1. **1.Do you work with personal or sensitive data in your research?** *

*Mark only one oval.*


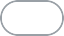
 Yes *Skip to question 25*


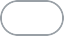
 No *Skip to question 26*

If yes, do you use data anonymization techniques before analysis?

1. **If yes, do you use data anonymization techniques before analysis?** *

*Mark only one oval.*


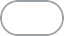
 Always
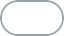
 Sometimes
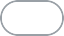
 Rarely
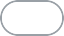
 Never


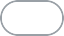
 Not Applicable

Are you aware of the data protection laws relevant to your research?

1. **2.Are you aware of the data protection laws relevant to your research?** *

*Mark only one oval.*


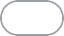
 Yes, very aware
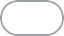
 Somewhat aware
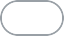
 Vaguely aware
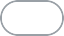
 Not aware

Section 8: Use of Technology in Ethics Review

1. **1.Has your institution implemented an online platform for ethics submissions?** *

*Mark only one oval.*


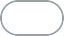
 Yes *Skip to question 28*


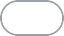
 No *Skip to question 29*


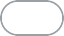
 Not Sure *Skip to question 29*

If yes, how would you rate the online ethics submission system?

1. **If yes, how would you rate the online ethics submission system?** *

*Mark only one oval.*


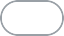
 Very Poor
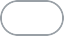
 Poor Neutral Good Excellent

Would you prefer digital or in-person support when submitting to ethics committees?

1. **2.Would you prefer digital or in-person support when submitting to ethics** *

committees?

*Mark only one oval.*

Digital (email, chat, portal) In-person consultation A combination of both No preference

Section 9: Training and Resources

1. **1.Have you ever attended a workshop or seminar focused on research ethics?** *

*Mark only one oval.*

Yes No

1. **2.Would you participate in additional training if made available?** *

*Mark only one oval.*

Yes No Maybe

1. **3.Preferred format for research ethics training:** *

*Mark only one oval.*

Online self-paced module Live online workshop In-person session

Written guides/manuals Not interested

Section 10: Future Outlook

1. **1.Do you believe ethical standards will become more strict in the next 5 years?** *

*Mark only one oval.*

Yes No

Not Sure

1. **2.Would you support the integration of AI tools to assist in ethics reviews (e.g.,** *

ﬂagging consent issues, automation)?

*Mark only one oval.*

Strongly Disagree Disagree

Neutral Agree

Strongly Agree

Section 11: Improving Ethics Awareness and Compliance

1. **1.What would help improve researchers’ awareness and compliance with ethical** *

review procedures? (Choose more than one answer)

*Tick all that apply.*

Mandatory training on research ethics

Clear and simpliﬁed submission guidelines Regular workshops or seminars

Online tutorials or orientation modules Institutional support or mentoring

Access to ethics committee consultation

Inclusion of ethics review in research curriculum Faster review and feedback process

Awareness campaigns or information drives

Section 12: Knowledge of Research Ethics.

1. **1.What do you understand by research ethics? (Open-ended)** *
2. **2.Which of the following do you think the a principle of research ethics ?** *

*Mark only one oval.*

Integrity

Respect for persons Beneﬁcence

Justice

All that apply

1. **3.Are you aware of any penalties for research misconduct?** *

*Mark only one oval.*

Yes No

Section 13: Awareness of Penalties for Research Misconduct.

1. **1.What penalties for research misconduct are you aware of? (Choose more than** *

one answer)

*Tick all that apply.*

Warning letters

Mandatory Ethics Training

Withdrawal of Published Papers

Rejection of Future Research Proposals Suspension from Research Activities Loss of Funding or Grants

Termination of Employment Legal Penalties (ﬁnes, etc.) Criminal Charges

All that apply

1. **2.How serious do you think the consequences of research misconduct are?** *

*Mark only one oval.*

Not serious at all

*Skip to section 24 (****Thank you very much for participating in this survey. We sincerely appreciate your time and effort. Your valuable contribution will greatly support us in achieving the goals of this study and enhancing the quality of the results.*** *)*

Slightly serious

*Skip to section 24 (****Thank you very much for participating in this survey. We sincerely appreciate your time and effort. Your valuable contribution will greatly support us in achieving the goals of this study and enhancing the quality of the results.*** *)*

Moderately serious

*Skip to section 24 (****Thank you very much for participating in this survey. We sincerely appreciate your time and effort. Your valuable contribution will greatly support us in achieving the goals of this study and enhancing the quality of the results.*** *)*

Very serious

*Skip to section 24 (****Thank you very much for participating in this survey. We sincerely appreciate your time and effort. Your valuable contribution will greatly support us in achieving the goals of this study and enhancing the quality of the results.*** *)*

Extremely serious

*Skip to section 24 (****Thank you very much for participating in this survey. We sincerely appreciate your time and effort. Your valuable contribution will greatly support us in achieving the goals of this study and enhancing the quality of the results.*** *)*

"Thank you for your time and interest. We fully respect your decision not to participate in this survey."

1. **"Thank you for your time and interest. We fully respect your decision not to** *

participate in this survey."

*Mark only one oval.*

Goodbye

Thank you very much for participating in this survey. We sincerely appreciate your time and effort. Your valuable contribution will greatly support us in achieving the goals of this study and enhancing the quality of the results.

This content is neither created nor endorsed by Google.

[Forms](https://www.google.com/forms/about/?utm_source=product&utm_medium=forms_logo&utm_campaign=forms)
